# Longitudinal trajectory of acidosis and mortality in acute kidney injury requiring continuous renal replacement therapy

**DOI:** 10.1186/s12882-022-03047-4

**Published:** 2022-12-26

**Authors:** Jinwoo Lee, Seong Geun Kim, Donghwan Yun, Min Woo Kang, Yong Chul Kim, Dong Ki Kim, Kook-Hwan Oh, Kwon Wook Joo, Yon Su Kim, Ho Seok Koo, Seung Seok Han

**Affiliations:** 1 Department of Internal Medicine, Seoul National University College of Medicine 103 Daehakro, Jongno-gu, Seoul, 03080 South Korea; 2 Department of Internal Medicine, Inje University College of Medicine, Busan, South Korea

**Keywords:** Acute kidney injury, Acidosis, Continuous renal replacement therapy, Inflammation, Mortality

## Abstract

**Background:**

Acidosis frequently occurs in severe acute kidney injury (AKI), and continuous renal replacement therapy (CRRT) can control this pathologic condition. Nevertheless, acidosis may be aggravated; thus, monitoring is essential after starting CRRT. Herein, we addressed the longitudinal trajectory of acidosis on CRRT and its relationship with worse outcomes.

**Methods:**

The latent growth mixture model was applied to classify the trajectories of pH during the first 24 hours and those of C-reactive protein (CRP) after 24 hours on CRRT due to AKI (*n* = 1815). Cox proportional hazard models were used to calculate hazard ratios of all-cause mortality after adjusting multiple variables or matching their propensity scores.

**Results:**

The patients could be classified into 5 clusters, including the normally maintained groups (1st cluster, pH = 7.4; and 2nd cluster, pH = 7.3), recovering group (3rd cluster with pH values from 7.2 to 7.3), aggravating group (4th cluster with pH values from 7.3 to 7.2), and ill-being group (5th cluster, pH < 7.2). The pH clusters had different trends of C-reactive protein (CRP) after 24 hours; the 1st and 2nd pH clusters had lower levels, but the 3rd to 5th pH clusters had an increasing trend of CRP. The 1st pH cluster had the best survival rates, and the 3rd to 5th pH clusters had the worst survival rates. This survival difference was significant despite adjusting for other variables or matching propensity scores.

**Conclusions:**

Initial trajectories of acidosis determine subsequent worse outcomes, such as mortality and inflammation, in patients undergoing CRRT due to AKI.

**Supplementary Information:**

The online version contains supplementary material available at 10.1186/s12882-022-03047-4.

## Introduction

Acute kidney injury (AKI) is a critical factor in increasing the mortality of critically ill patients admitted to the intensive care unit (ICU) [1–5]. Continuous renal replacement therapy (CRRT) is a rescue measure for patients with both unstable vital signs and severe AKI. The number of AKI cases requiring CRRT has increased to more than 150,000 in the United States over the past few decades [6]. Despite advances in CRRT technology, the patient outcomes of CRRT due to AKI are still worse [4, 5, 7–10]. Although guidelines exist for CRRT implementation [11–13], CRRT-related complications can occur, and initiating CRRT does not always guarantee a survival advantage, which indicates the importance of an individualized approach [14–17].

Metabolic acidosis is an important feature in severe AKI [7, 18]. This pathologic condition is attributable to decreased excretion of nonvolatile acids via urination and decreased renal synthesis of bicarbonates [19]. CRRT can successfully control metabolic acidosis, and exogenous bicarbonates may be added during CRRT. Nevertheless, incomplete correction frequently occurs, perhaps because of less correction by CRRT or high production of acids by patient aggravation. Previous studies have investigated the correlation between pH and mortality in patients receiving CRRT [7, 18], and initial pH on CRRT was related to subsequent high mortality.

To date, no studies have considered the acidosis trend and its relationship with outcomes after starting CRRT. Herein, we aimed to address this issue by clustering the first 24-hour trajectories of pH on CRRT, and identified that certain trajectory groups had high mortality outcomes despite adjusting multiple variables or matching propensity scores. Furthermore, the pH trends determined the subsequent trend of systemic inflammation evaluated with high sensitivity C-reactive protein (CRP) levels, which might contribute to mortality differences.

## Methods

### Patients and data collection

**Fig. 1:**
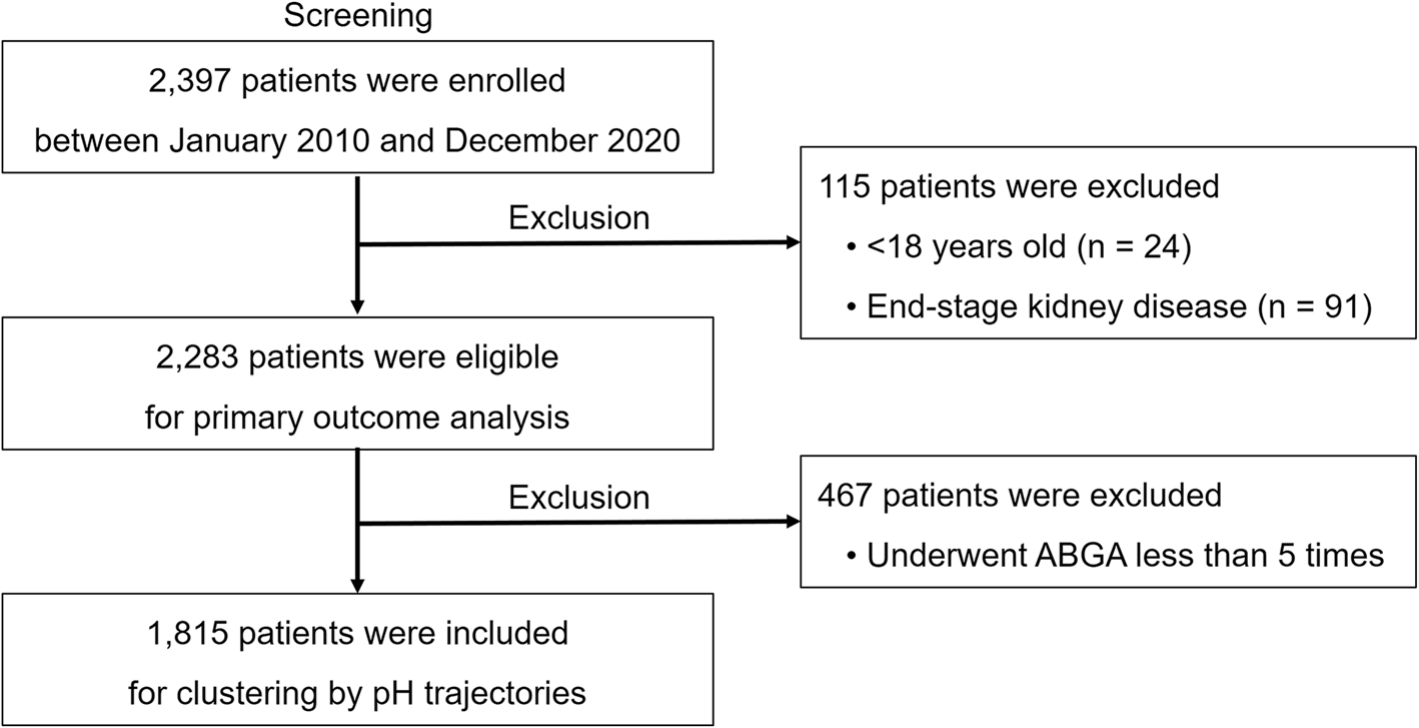
Flow diagram for study subjects. ABGA, artery blood gas analysis

A total of 2397 patients undergoing CRRT due to AKI were retrospectively reviewed at Seoul National University Hospital from June 2010 to December 2020. Patients who were aged < 18 years (*n* = 24) and who had end-stage kidney disease at the time of initiating CRRT (*n* = 91) were excluded. Patients who underwent arterial blood gas analysis less than 5 times in the first 24 hours after the initiation of CRRT (*n* = 467) were also excluded. Accordingly, 1815 patients were included in the analyses (Fig. 1). The study design was approved by the institutional review board of Seoul National University Hospital (No. H-2110-085-1262) and compiled with the Declaration of Helsinki. Informed consent was waived under approval.

Baseline data were collected, such as age, sex, weight, cause of AKI (e.g., septic and nonseptic), ICU division, use of inotropes, application of mechanical ventilation, type of central catheter, setting of CRRT (e.g., blood flow rate, target dose, and ultrafiltration), use of bicarbonate ampoules, and anuria status. The severity of illness was evaluated using the Charlson comorbidity index (CCI) [20], sequential organ failure assessment (SOFA) [21], and acute physiology assessment and chronic health evaluation (APACHE) II [22]. The primary outcome was all-cause mortality after starting CRRT. The high-sensitivity CRP levels were measured after 24 hours until 72 hours to determine the correlation with the first 24-hour pH trends.

### Statistical analysis

Baseline characteristics are described as proportions and means ± standard deviations when categorical and continuous variables were normally distributed and as medians with interquartile ranges when they were not normally distributed. The normality of the distribution was analyzed using the Kolmogorov–Smirnov test. A chi-square test or Fisher’s exact test was used to compare categorical variables. Student’s t test or the Mann–Whitney U test was used for continuous variables with or without a normal distribution, respectively. ANOVA with post hoc analysis was used to evaluate the difference in baseline characteristics. The latent growth mixture model was applied to classify the trajectory of change in pH and CRP. To reflect the goodness of fit of the linear-linear latent growth mixture model, the Akaike information criterion, Bayesian information criterion, and sample-adjusted Bayesian information criterion were used. Cox proportional hazard models with and without stepwise adjustment of multiple variables were used to calculate the hazard ratio (HR) of mortality outcomes. We tested the proportional hazard assumption using the Schoenfeld test. Kaplan–Meier survival curves were drawn, and differences in the curves were determined using a log-rank test. Because many baseline parameters differed between groups, propensity score-based matching with inverse probability treatment weighting was additionally performed. All baseline variables were used to calculate propensity scores. A two-tailed *P* value less than 0.05 was considered statistically significant. All statistical analyses were performed using R software (version 4.1.2; R core team, Vienna, Austria).

## Results

### Baseline characteristics

The baseline characteristics are presented in Table 1. The mean age was 64 ± 15 years, and 62.8% of the patients were male. The proportion of septic AKI was 47.0, and 51.7% of the patients were hospitalized in the medical ICU. Half of the patients used inotropes and approximately 80% of patients were supported by mechanical ventilations. Half of the patients used femoral catheters as vascular access. Their SOFA and APACHE II scores were 11.9 ± 3.7 and 26.2 ± 7.8, respectively. In total, pCO_2_ was 35.6 ± 12.5 mmHg. Partial pressure of carbon dioxide (pCO_2_) was significantly higher in the 5th cluster compared to the 1st cluster (Table S2), indicating that respiratory acidosis was superimposed or respiratory compensation was not performed in the 5th cluster. Anion gap and serum lactate level were 17.7 ± 7.9 mmol/l and 6.4 ± 5.1 mmol/l, respectively. Serum anion gap and lactate were highest in the 5th cluster among clusters. However, in contrary to pCO_2_, serum anion gap and lactate showed abnormal levels even in the 1st cluster, suggesting that nonvolatile acids, such as lactate and phosphate, accumulated in all clusters (Table S2).

**Table 1:** Baseline characteristics of the patients

Variables	Total (*n* = 1815)	1st cluster (*n* = 575)	2nd cluster (*n* = 748)	3rd cluster (*n* = 186)	4th cluster (*n* = 188)	5th cluster (*n* = 118)	*P* for trend
Age (years)	64.1 ± 14.9	63.6 ± 15.7	64.0 ± 15.0	63.8 ± 14.9	65.1 ± 14.0	62.0 ± 15.1	0.567
Male (%)	62.8	61.6	61.8	58.6	68.6	72.9*	0.072
Weight (kg)	61.8 ± 13.3	61.6 ± 12.9	61.8 ± 13.0	60.3 ± 12.5	63.3 ± 13.2	66.0 ± 17.1^†^	0.046
Septic AKI (%)	47	41.7	45.2	58.2^‡^	54.0*	53.2*	< 0.001
ICU division (%)							0.069
MICU	51.7	47.8	51.1	58.6^‡^	57.4^†^	54.2^‡^	
SICU	19.1	21.2	19.4	11.8	18.1	19.5	
CPICU	12.7	17.2	14.6	4.3	6.4	1.7	
EICU	16.3	13.2	14.8	24.7	18.1	24.6	
DICU	0.3	0.5	0.1	0.5	0	0	
Inotropics use (%)	48.9	49.9	46.8	52.2	48.9	51.7	0.892
Mechanical ventilator (%)	79.4	72.2	80.5^‡^	86.0^‡^	85.6^‡^	88.1^‡^	< 0.001
Catheter (%)							< 0.001
Intrajugular	37.6	43.3	36.2*	33.3*	35.1	29.7*	
Femoral	52.2	48.3	53.6	53.8	52.7	59.3	
Others	10.1	8.3	10.2	12.9	12.2	11	
Blood flow rate (ml/min)	110.9 ± 24.5	110.0 ± 25.2	111.4 ± 25.6	113.8 ± 25.7	112.7 ± 24.2	110.1 ± 23.8	0.986
Target dose (ml/kg/hr)	42.2 ± 15.2	41.3 ± 15.2	41.3 ± 14.7	43.6 ± 14.4	42.3 ± 13.3	41.8 ± 14.1	0.988
Target UF (ml/d)	0 (0–500)	0 (0–500)	0 (0–1000)	0 (0–500)^†^	0 (0–500)	0 (0–500)^‡^	< 0.001
Bicarbonate use (ample/d)	0 (0–4)	0 (0–0)	0 (0–4)^‡^	4 (0–8)^‡^	4 (0–8)^‡^	4 (0–8)^‡^	< 0.001
Anuria (%)	27.9	23.3	24.9	38.3^†^	37.6^†^	35.5*	0.002
CCI score	3.3 ± 2.3	3.2 ± 2.2	3.4 ± 2.3	3.4 ± 2.4	3.5 ± 2.5	3.1 ± 2.3	0.849
SOFA score	11.9 ± 3.7	11.1 ± 3.6	11.9 ± 3.5^‡^	12.2 ± 3.7^‡^	13.0 ± 3.1^‡^	13.2 ± 3.4^‡^	< 0.001
APACHE II score	26.2 ± 7.8	23.6 ± 6.8	25.5 ± 7.3^‡^	28.8 ± 8.1^‡^	29.1 ± 7.0^‡^	30.9 ± 6.7^‡^	< 0.001

*AKI* Acute kidney injury, *ICU* Intensive care unit, *MICU* Medical intensive care unit, *SICU* Surgical intensive care unit, *CPICU* Cardio-pulmonary intensive care unit, *EICU* Emergency intensive care unit, *DICU* Disaster intensive care unit for covid-19 infection, *UF* Ultrafiltration, *CCI* Charlson comorbidity index, *SOFA* Sequential organ failure assessment, *APACHE* Acute physiologic and chronic health evaluation

### Clustering of acidosis trajectories

**Fig. 2:**
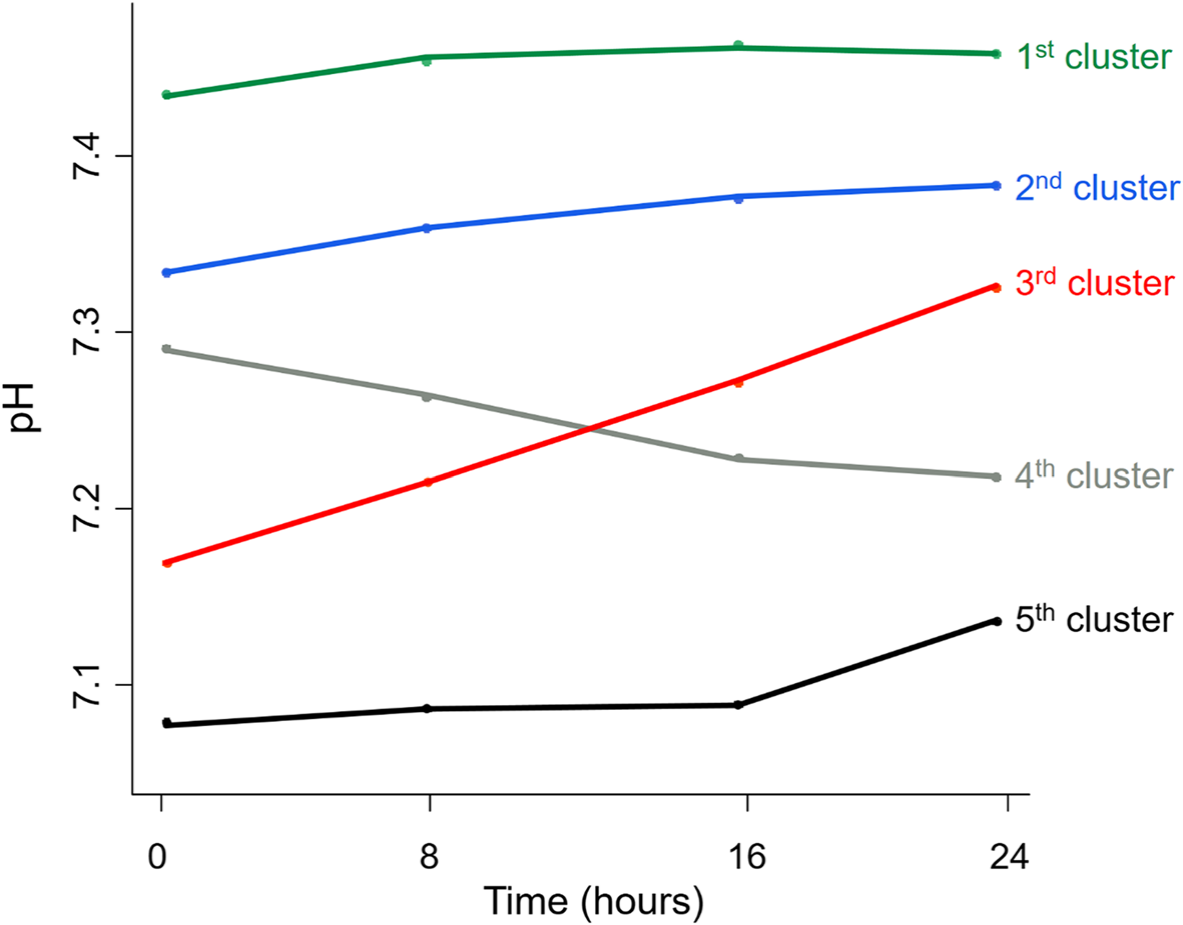
Clustering of pH trajectories during the first 24 hours after starting continuous renal replacement therapy. 1st cluster, consistently normal pH levels on CRRT; 2nd cluster, suboptimal pH trajectory with initial pH 7.3, and gradually approached 7.4; 3rd cluster, recovering acidosis from pH 7.2 to 7.3; 4th cluster, aggravating tendency of acidosis from pH 7.3 to 7.2; 5th cluster, uncorrected pH trajectory less than 7.2

Patients were classified into 5 clusters based on the distinctive trends of pH (Fig. 2). The 1st cluster, accounting for 31.7% of patients, showed consistently normal pH levels on CRRT. The 2nd cluster, accounting for 41.2% of patients, had suboptimal pH trajectory with initial pH 7.3, and gradually approached 7.4. The 3rd cluster, accounting for 10.2% of patients, had recovering acidosis from pH 7.2 to 7.3. The 4th cluster (10.4% of patients) had an aggravating tendency of acidosis from pH 7.3 to 7.2 in spite of performing CRRT. The 5th cluster (6.5% of patients) also had uncontrolled pH trajectory less than 7.2 despite implementation of CRRT.

The variables of age, sex, ICU division, inotrope use, blood flow rate, target dose, and CCI score were not different among the 5 clusters (Table 1). Septic AKI was most common in the 3rd cluster, explaining 58.2% of the total number. The SOFA and APACHE II scores tended to increase in the groups with low pH values, such as the 4th and 5th clusters. Mechanical ventilation was applied in 72.2% of the 5th cluster.

### Association between acidosis trajectory and survival

**Fig. 3:**
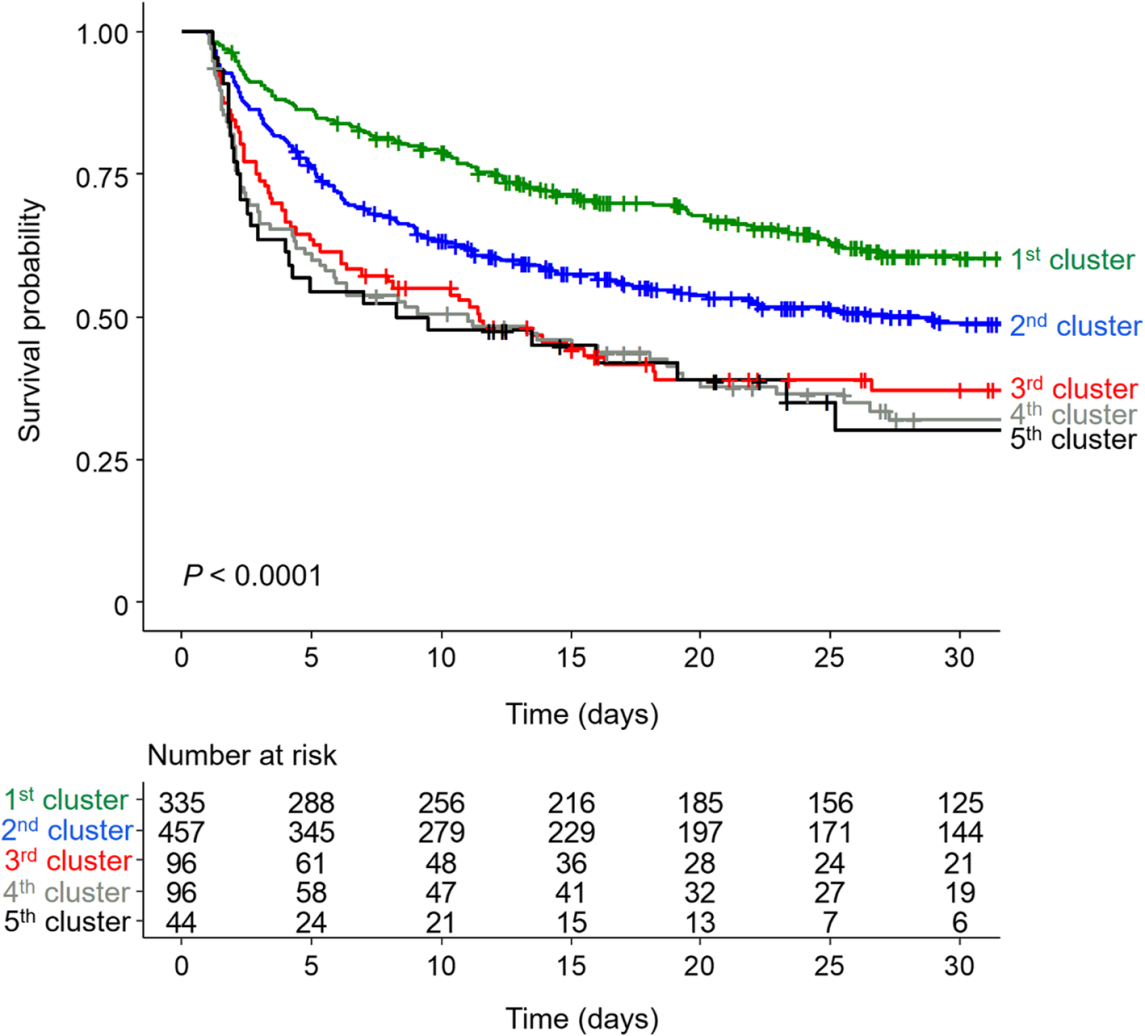
Kaplan–Meier survival curves of pH clusters for all-cause mortality. 1st cluster, consistently normal pH levels on CRRT; 2nd cluster, suboptimal pH trajectory with initial pH 7.3, and gradually approached 7.4; 3rd cluster, recovering acidosis from pH 7.2 to 7.3; 4th cluster, aggravating tendency of acidosis from pH 7.3 to 7.2; 5th cluster, uncorrected pH trajectory less than 7.2

During a median follow-up period of 10 days (interquartile range, 3–28 days), 1193 patients (65.7%) died. The mortality incidence was 26.7 deaths per 1000 patient-days. The all-cause mortality rates were 44.3, 55.7, 74.2, 78.2 and 82.2% from the 1st cluster to the 5th cluster, respectively (*P* < 0.001). Fig. 3 shows Kaplan–Meier survival curves of 5 clusters, and their curves were separated (*P* < 0.001). The mortality risk increased from the 1st cluster to the 5th cluster, irrespective of adjusting for multiple variables (Table 2). Because several baselines differed between the clusters, we matched propensity scores with two methods. Despite matching propensity scores, the mortality rates were different, similar to the above results (Table 3).

**Table 2:** Risk of mortality according to the acidosis trajectories

Models	Groups	HR (95% CI)	*P*
Model 1	1st cluster	Reference	
	2nd cluster	1.433 (1.220–1.683)	< 0.001
	3rd cluster	2.291 (1.816–2.890)	< 0.001
	4th cluster	2.700 (2.152–3.389)	< 0.001
	5th cluster	3.037 (2.254–4.093)	< 0.001
Model 2	1st cluster	Reference	
	2nd cluster	1.430 (1.217–1.680)	< 0.001
	3rd cluster	2.297 (1.820–2.899)	< 0.001
	4th cluster	2.716 (2.164–3.411)	< 0.001
	5th cluster	3.057 (2.269–4.120)	< 0.001
Model 3	1st cluster	Reference	
	2nd cluster	1.240 (1.010–1.521)	0.039
	3rd cluster	1.542 (1.131–2.103)	0.006
	4th cluster	1.842 (1.370–2.476)	< 0.001
	5th cluster	1.802 (1.208–2.686)	0.003

Model 1: Unadjusted

Model 2: Adjusted for age and sex

Model 3: Model 2 plus weight, septic AKI, ICU division, anuria, CCI, SOFA and APACHE II

*HR *Hazard ratio, *CI *Confidence interval

**Table 3:** Comparison of mortality after matching propensity scores

Matching method	Groups	HR (95% CI)	*P*
IPTW-logistic	1st cluster	Reference	
	2nd cluster	1.120 (1.014–1.415)	0.034
	3rd cluster	1.673 (1.217–2.299)	0.002
	4th cluster	2.327 (1.781–3.040)	< 0.001
	5th cluster	2.138 (1.550–2.948)	< 0.001
IPTW-XGboost	1st cluster	Reference	
	2nd cluster	1.193 (1.011–1.408)	0.037
	3rd cluster	1.640 (1.165–2.309)	0.005
	4th cluster	2.348 (1.822–3.025)	< 0.001
	5th cluster	1.925 (1.328–2.789)	0.001

*HR* Hazard ratio, *CI* Confidence interval, *IPTW* Inverse probability treatment weighting, *XG boost* Extreme gradient boosting

### Association between acidosis and inflammation trajectories

**Fig. 4:**
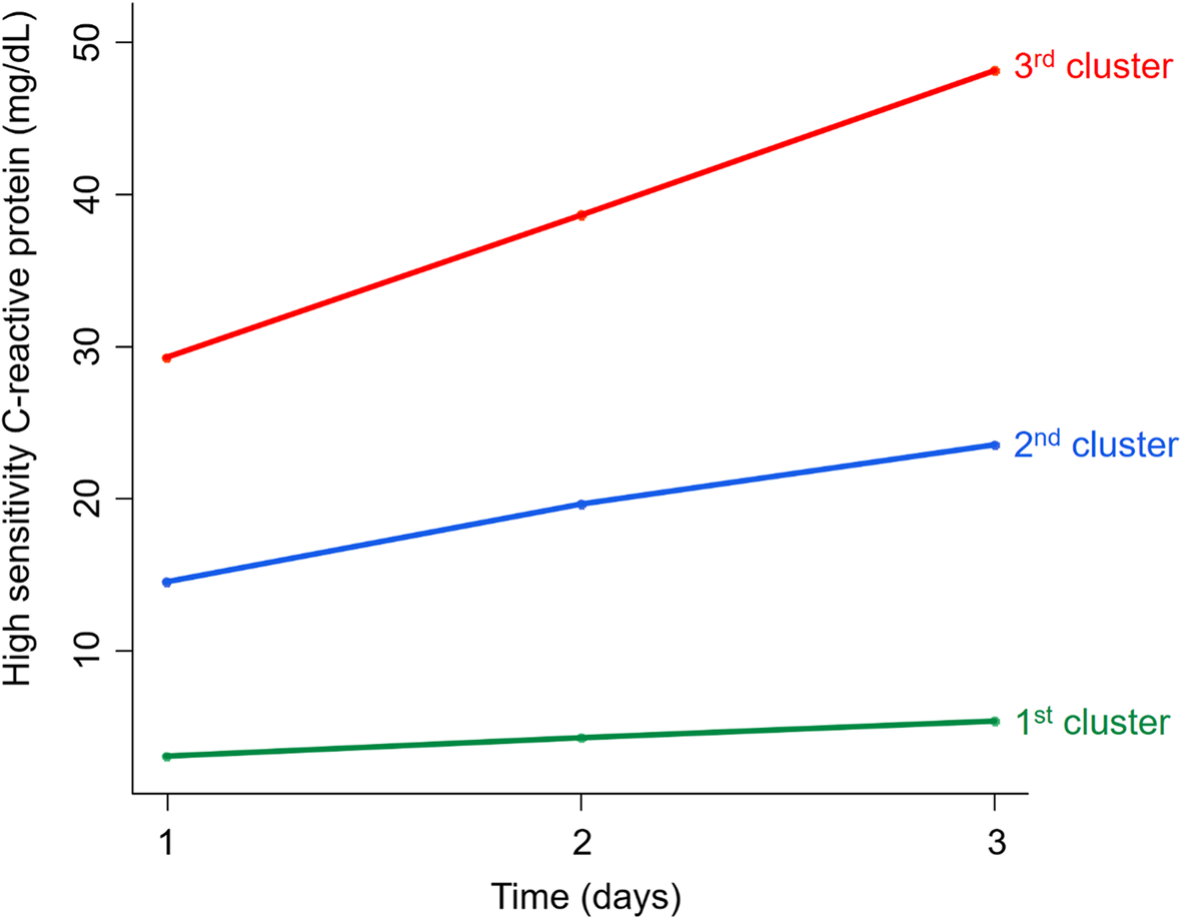
Clustering of trajectories in high sensitivity C-reactive protein between 24 and 72 hours after starting continuous renal replacement therapy. 1st cluster, stationary trend of CRP less than 10 mg/dL; 2nd cluster, increasing trend of CRP between 15 and 20 mg/dL; 3rd cluster, increasing trend of CRP between 30 and 50 mg/dL

**Fig. 5:**
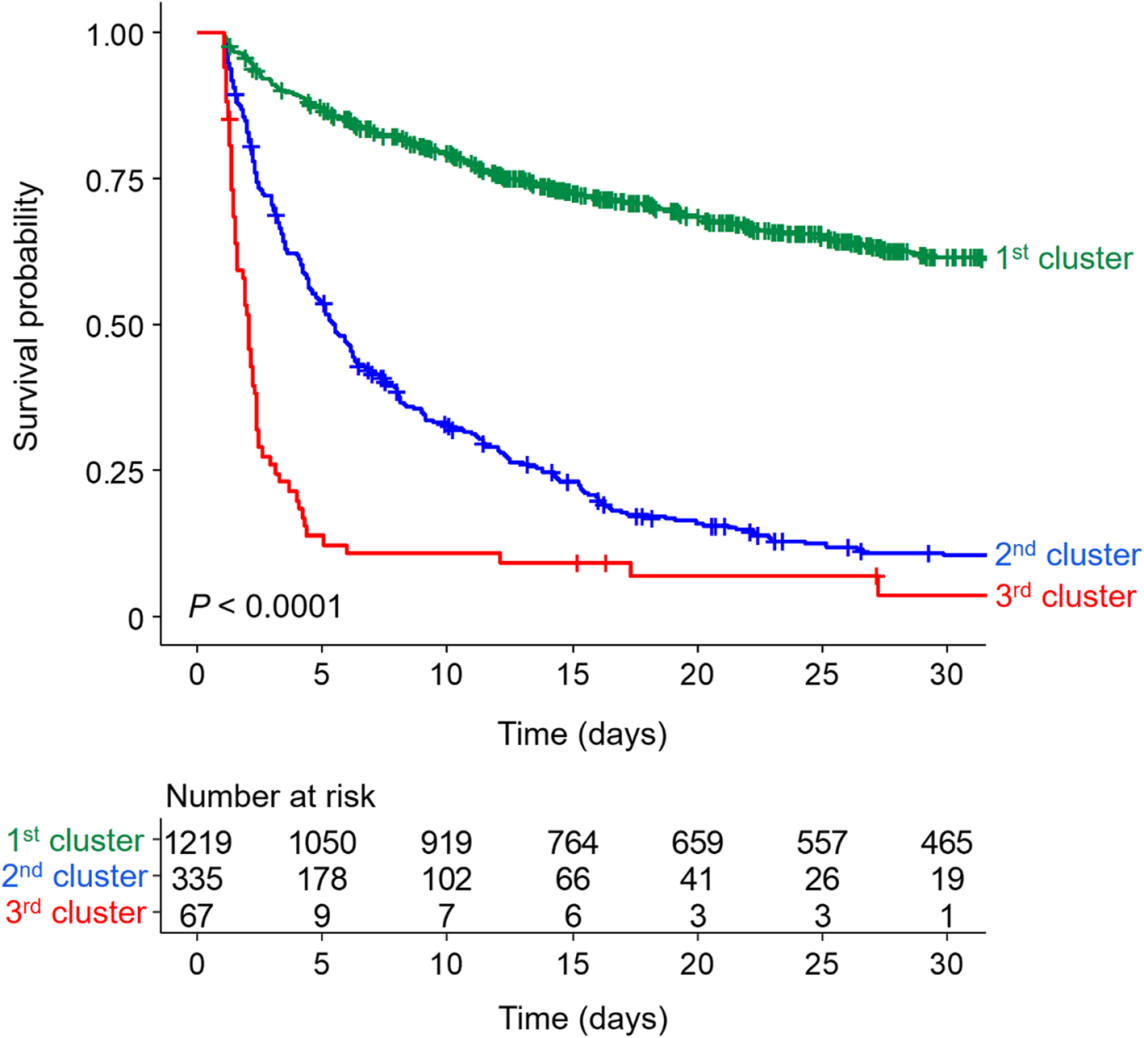
Kaplan–Meier survival curves of high-sensitivity C-reactive protein clusters for all-cause mortality. 1st cluster, stationary trend of CRP less than 10 mg/dL; 2nd cluster, increasing trend of CRP between 15 and 20 mg/dL; 3rd cluster, increasing trend of CRP between 30 and 50 mg/dL

**Fig. 6:**
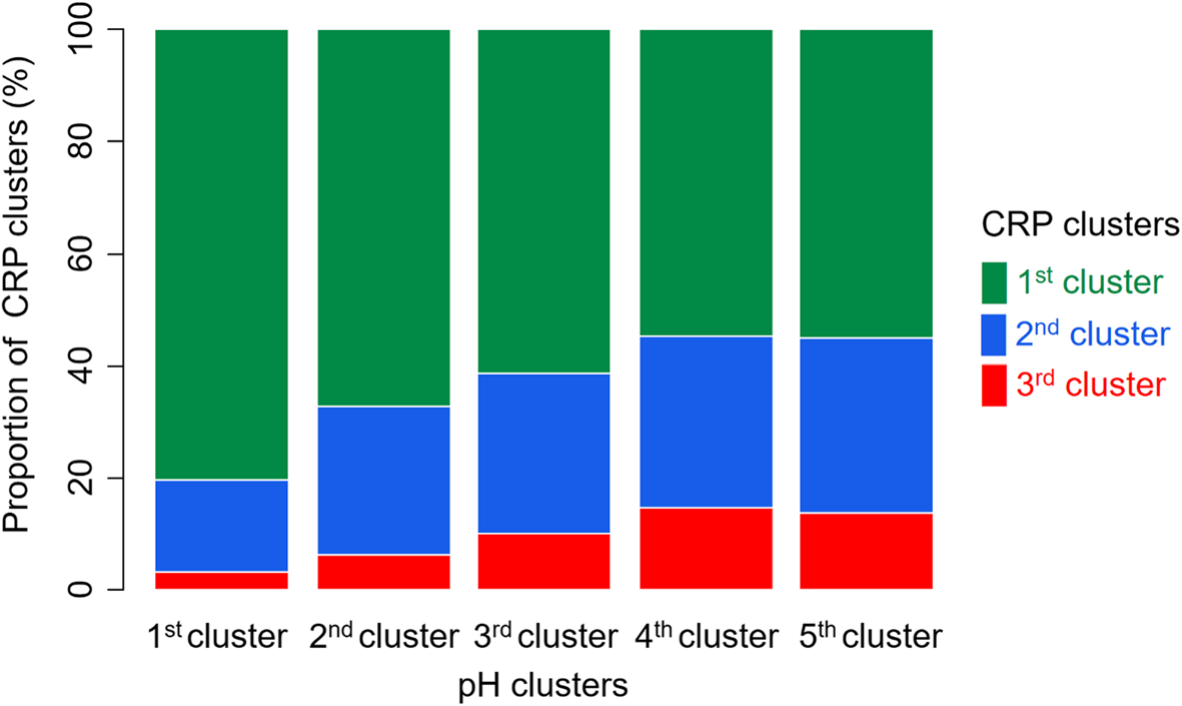
Distribution of high-sensitivity C-reactive protein (CRP) clusters according to pH clusters. 1st cluster, stationary trend of CRP less than 10 mg/dL; 2nd cluster, increasing trend of CRP between 15 and 20 mg/dL; 3rd cluster, increasing trend of CRP between 30 and 50 mg/dL

To evaluate the subsequent inflammatory status, clustering was performed using the CRP values between 1 and 3 days on CRRT. A total of 3 clusters were identified by distinctive trends of CRP (Fig. 4). The 1st cluster had a stationary trend of CRP, and the 2nd and 3rd clusters had an increasing trend of CRP. The Kaplan–Meier curves showed the best survival rate in the 1st cluster. In contrast, survival rate was worst in 3rd cluster (Fig. 5). As the pH trajectory approached the normal value of 7.4, the proportion of patients with a low CRP trajectory increased (Fig. 6). The results suggest that the association between acidosis trajectories and mortality was partly attributable to subsequent inflammatory status.

## Discussion

Since AKI patients who require CRRT are in critical condition, clinicians need to consider the patient status, including vital signs, biochemical results, imaging tests, and medical history. Among these, acidosis is one that reflects hemodynamics, respiration, and tissue oxygenation at once [23, 24]. Herein, acidosis trajectories were associated with subsequent mortality and inflammatory trends after starting CRRT. The relationship between acidosis trajectories and outcomes was independent of other variables; thus, monitoring both initial and subsequent trends of acidosis is important in this patient subset.

The detrimental effect of acidosis on patient outcome has been documented, particularly in chronic kidney disease [25–27]. There is a consensus that metabolic acidosis leads to insulin resistance, breakdown in skeletal muscle mass, and cardiovascular complications in addition to progression of kidney disease [28–30]. Observational studies have shown that metabolic acidosis is associated with risks of doubling of serum creatinine and all-cause mortality compared to the counterpart normal status [25, 29, 31, 32]. Small sample-sized clinical trials on alkali supplementation and dietary intervention have demonstrated the beneficial effect of correcting metabolic acidosis on preserving kidney function in patients with chronic kidney disease [33–35].

Similar to chronic kidney disease, metabolic acidosis may be associated with poorer outcomes in AKI patients [36–39]. Acidosis would be both the cause and result of AKI [40]. Despite the complex relationship between acidosis and AKI, a linear relationship was observed between baseline pH and outcome in most studies [7, 18]. Here, pH trajectories after starting CRRT were diverse, and some cases could not be recovered from acidosis despite the same protocol on CRRT. Accordingly, the relationship between acidosis trajectories and mortality outcomes was prominent, which indicates that the acidosis trend, in addition to initial acid-base status, is also important for determining the patient outcomes.

The process of acidosis begins with the formation of free radicals, which leads to oxidative stress and results in endothelial dysfunction; finally, cytokines are released that make it difficult to maintain an appropriate blood pressure level [41–44]. Furthermore, acidosis causes malnutrition, which is primarily related to poor survival in patients with septic shock [45–47]. This pathophysiology of metabolic acidosis in AKI is similar to that of CKD, where persistent renal acidosis leads to cardiovascular complications, bone mineral disease, and CKD progression [25–27, 29, 30, 48, 49].

The present study has strengths, such as no missing values and concrete statistical analyses. Nonetheless, there are certain limitations to be discussed. First, because the study design was retrospective in nature, the results could not determine causality between acidosis trajectories and outcomes. Selection bias and residual confounding factors might exist, although we used matching methods to overcome them. Second, other pH-related biochemical parameters, such as pCO_2_, anion gap, and lactic acid, were not traced, making it difficult to interpret the independent relationship with outcomes. Nevertheless, we collected information such as baseline pCO_2_, anion gap, and lactate to differentiate the early causes of acidosis in each pH cluster. Finally, time-varying ICU cares, including shifts in catheter site, changes in CRRT settings, and inotropic dosage were not considered in the analysis.

In conclusion, acidosis trajectories determine subsequent worse outcomes, such as high mortality and systemic inflammatory response in patients starting CRRT due to AKI. Accordingly, precise monitoring of acidosis on CRRT may be helpful to predict patient outcomes. However, randomized clinical trial is needed to determine whether pH correction during CRRT improves the survival rate in patients with specific pH groups and to overcome the limitation of retrospective nature. Future trials will address other clinical outcomes, such as renal recovery and ventilator weaning. Hopefully, the present results will be a conceptual rationale for clinical trials with acidosis correction.

## Supplementary Information

**Additional file 1:Table S1.** Baseline patient characteristics after extreme gradient boosting model with inverse probability treatment weighting-based propensity scores matching.

**Additional file 2:Table S2.** Baseline serum pH determinants according to the pH clusters.

## Data Availability

The datasets used and/or analyzed during the current study are available from the corresponding author upon reasonable request.

## Abbreviations

AKI: Acute kidney injury
ICU: Intensive care unit
CRRT: continuous renal replacement therapy
CRP: C-reactive protein
CCI: Charlson comorbidity index
SOFA: sequential organ failure assessment
APACHE: acute physiology assessment and chronic health evaluation
HR: hazard ratio
