# Supplementary material for: Longitudinal trajectory of acidosis and mortality in acute kidney injury requiring continuous renal replacement therapy
Source: BMC Nephrol. 2022 Dec 26;23:411. doi: 10.1186/s12882-022-03047-4 (PMC9792158; doi:10.1186/s12882-022-03047-4)
Supplement: Supplementary file 1 — Additional file 1:Table S1. Baseline patient characteristics after extreme gradient boosting model with inverse probability treatment weighting-based propensity scores matching. [file 12882_2022_3047_MOESM1_ESM.docx]

Table S1. Baseline patient characteristics after extreme gradient boosting model with inverse probability treatment weighting-based propensity scores matching

| Variables | 1^st^ cluster  (n = 1,533) | 2^nd^ cluster  (n = 1,466) | 3^rd^ cluster  (n = 1,118) | 4^th^ cluster  (n = 1,198) | 5^th^ cluster  (n = 803) | *P* |
| --- | --- | --- | --- | --- | --- | --- |
| Age (years) | 64.0 ± 15.1 | 64.0 ± 14.8 | 65.0 ± 13.8 | 64.0 ± 14.4 | 64.0 ± 13.5 | 0.951 |
| Male (%) | 61.4 | 62.5 | 62.3 | 63.7 | 71.9 | 0.446 |
| Weight (kg) | 61.8 ± 13.0 | 61.7 ± 13.0 | 62.0 ± 12.1 | 62.4 ± 13.4 | 62.6 ± 14.5 | 0.970 |
| Septic AKI (%) | 46.1 | 45.4 | 55.6 | 52.7 | 53.9 | 0.254 |
| ICU division (%) |  |  |  |  |  | 0.381 |
| MICU | 50.2 | 51.6 | 54.2 | 52.5 | 64.3 |  |
| SICU | 18.9 | 19.1 | 14.1 | 19.1 | 14.9 |  |
| CPICU | 13.9 | 13.1 | 10.2 | 6.9 | 4.8 |  |
| EICU | 16.6 | 16.0 | 20.4 | 21.5 | 16.0 |  |
| DICU | 0.4 | 0.1 | 1.0 | 0 | 0 |  |
| Inotropic use (%) | 50.9 | 48.6 | 49.4 | 51.2 | 46.7 | 0.925 |
| Mechanical ventilator (%) | 78.7 | 80.3 | 81.7 | 80.6 | 88.1 | 0.482 |
| Catheter (%) |  |  |  |  |  | 0.818 |
| Intrajugular | 37.9 | 37.3 | 32.8 | 37.7 | 30.2 |  |
| Femoral | 52.7 | 52.8 | 54.0 | 50.5 | 58.6 |  |
| Others | 9.5 | 9.9 | 13.1 | 11.8 | 11.2 |  |
| Blood flow rate (ml/min) | 110.7 ± 24.7 | 110.9 ± 25.1 | 113.5 ± 26.0 | 113.0 ± 25.9 | 109.8 ± 24.4 | 0.708 |
| Target dose (ml/kg/hr) | 41.7 ± 14.8 | 41.5 ± 14.4 | 41.7 ± 13.3 | 41.5 ± 12.6 | 42.1 ± 12.8 | 0.995 |
| Target UF (ml/d) | 400 (0–1000) | 450 (0–1100) | 450 (0–1200) | 350 (0–800) | 250 (0–650) | 0.037 |
| Bicarbonate use (ample/d) | 2 (0–6) | 2 (0–6) | 3 (0–7) | 3 (0–8) | 4 (0–8) | 0.037 |
| Anuria (%) | 28.1 | 27.2 | 33.4 | 35.8 | 28.8 | 0.320 |
| CCI score | 3.3 ± 2.3 | 3.3 ± 2.2 | 3.4 ± 2.4 | 3.3 ± 2.3 | 3.3 ± 2.2 | 0.933 |
| SOFA score | 11.8 ± 3.6 | 11.8 ± 3.5 | 12.1 ± 3.4 | 12.3 ± 3.3 | 12.2 ± 3.5 | 0.591 |
| APACHE II score | 25.5 ± 7.3 | 25.8 ± 7.4 | 27.3 ± 7.5 | 27.0 ± 7.3 | 28.1 ± 6.6 | 0.007 |

- In propensity scores matching model with the IPTW-XGboost, matching for age, sex, weight, cause of AKI, ICU division, inotropic use, application of mechanical ventilator, type of catheter, blood flow rate, target dose, target ultrafiltration, bicarbonate use, presence of anuria, CCI, SOFA and APACHE II score.

- Abbreviations: AKI, acute kidney injury; ICU, intensive care unit; MICU, medical intensive care unit; SICU, surgical intensive care unit; CPICU, cardio-pulmonary intensive care unit; EICU, emergency intensive care unit; DICU, disaster intensive care unit for covid-19 infection; UF, ultrafiltration; CCI, Charlson comorbidity index; SOFA, sequential organ failure assessment; APACHE, acute physiologic and chronic health evaluation, IPTW, inverse probability treatment weighting; XGboost, extreme gradient boosting.
