# Supplementary material for: Longitudinal trajectory of acidosis and mortality in acute kidney injury requiring continuous renal replacement therapy
Source: BMC Nephrol. 2022 Dec 26;23:411. doi: 10.1186/s12882-022-03047-4 (PMC9792158; doi:10.1186/s12882-022-03047-4)
Supplement: Supplementary file 2 — Additional file 2:Table S2. Baseline serum pH determinants according to the pH clusters. [file 12882_2022_3047_MOESM2_ESM.docx]

Table S2. Baseline serum pH determinants according to the pH clusters

| Variables | Total  (n = 1,815) | 1^st^ cluster  (n = 575) | 2^nd^ cluster  (n = 748) | 3^rd^ cluster  (n = 186) | 4^th^ cluster  (n = 188) | 5^th^ cluster  (n = 118) | *P* |
| --- | --- | --- | --- | --- | --- | --- | --- |
| pCO_2_ (mmHg) | 35.6 ± 12.5 | 32.3 ± 8.9 | 35.6 ± 10.2 | 39.9 ± 17.9 | 37.5 ± 13.1 | 42.0 ± 21.7 | <0.001 |
| HCO_3_^–^ (mmol/L) | 18.3 ± 5.9 | 20.5 ± 5.5 | 18.6 ± 5.4 | 14.4 ± 5.3 | 17.6 ± 4.9 | 13.0 ± 5.9 | <0.001 |
| Anion gap | 17.7 ± 7.9 | 15.3 ± 5.9 | 17.2 ± 7.4 | 21.4 ± 9.0 | 18.6 ± 7.6 | 25.3 ± 10.1 | <0.001 |
| Lactate (mmol/L) | 6.4 ± 5.1 | 4.7 ± 4.2 | 6.1 ± 4.8 | 8.8 ± 5.3 | 7.9 ± 5.3 | 10.8 ± 5.6 | <0.001 |
